# Image segmentation with traveling waves in an exactly solvable recurrent neural network

**DOI:** 10.1073/pnas.2321319121

**Published:** 2025-01-03

**Authors:** Luisa H. B. Liboni, Roberto C. Budzinski, Alexandra N. Busch, Sindy Löwe, Thomas A. Keller, Max Welling, Lyle E. Muller

**Affiliations:** ^a^Department of Mathematics, Western University, London, ON N6A 3K7, Canada; ^b^Western Institute for Neuroscience, Western University, London, ON N6A 3K7, Canada; ^c^Western Academy for Advanced Research, Western University, London, ON N6A 3K7, Canada; ^d^Fields Lab for Network Science, Fields Institute, Toronto, ON M5T 3J1, Canada; ^e^Amsterdam Machine Learning Lab, University of Amsterdam, Amsterdam 1012 WP, The Netherlands; ^f^University of Amsterdam-Bosch Deep Learning Technologies Amsterdam Lab, University of Amsterdam, Amsterdam 1012 WP, The Netherlands

**Keywords:** image segmentation, recurrent neural networks, spatiotemporal dynamics, explainable AI, visual system

## Abstract

We study image segmentation using spatiotemporal dynamics in a recurrent neural network where the state of each unit is given by a complex number. We show that this network generates sophisticated spatiotemporal dynamics that can effectively divide an image into groups according to a scene’s structural characteristics. We then demonstrate a simple algorithm for object segmentation that generalizes across inputs ranging from simple geometric objects in grayscale images to natural images. Using an exact solution of the recurrent network’s dynamics, we present a precise description of the mechanism underlying object segmentation in the network dynamics, providing a clear mathematical interpretation of how the algorithm performs this task. Object segmentation across all images is accomplished with one recurrent neural network that has a single, fixed set of weights. This demonstrates the expressive potential of recurrent neural networks when constructed using a mathematical approach that brings together their structure, dynamics, and computation.

## Significance

Neural networks are powerful tools for processing visual inputs, but precisely how this processing is performed remains unclear. We introduce a recurrent neural network that can perform simple image segmentation while also being exactly mathematically solvable. This allows us to “open the box” to understand precisely how the internal connections within the network create visual computations in terms of a mathematical equation. This framework surpasses the standard level of understanding of how neural networks process visual input, creating an advance in “explainable AI”. These results may open a different path in machine learning powered by recurrent neural networks that allow precise mathematical insight, in addition to shedding light on the potential function of recurrent connections in biological visual systems.

Image segmentation is a fundamental task in computer vision. Whether finding regions of interest in medical images or highlighting specific objects, the ability to effectively divide an image into groups based on the structure in a scene can greatly facilitate image processing. Many techniques have been developed for image segmentation, from classical watershed (1) or active contour (2) algorithms to modern slot-based (3) and deep-learning approaches (4). Automated object segmentation represents a specific goal for image segmentation algorithms, in which pixels within the same object are grouped together. Finding objects in a data-driven manner allows dividing an input image into parts, opening up opportunities for further processing and semantic understanding.

Recent work in unsupervised image segmentation has utilized a special kind of autoencoder, where each node in the network has a state characterized by a complex number (5). Because the state of each node in this complex-valued network has both an amplitude and a phase, the intensity of each pixel can be encoded in the amplitude, and objects can be encoded in groups of nodes with similar phases. In terms of a physical system, groups of nodes with similar phases can be thought of as oscillators that synchronize when they are part of the same object. This physical analogy has been utilized in segmenting images in networks of spring-mass harmonic oscillators (6), chaotic maps (7, 8), and Kuramoto oscillators (9), where nodes that are part of the same object will synchronize to a phase that is unique from the phase of different objects.

In these previous works, segmentation occurs when all oscillators within an object synchronize on approximately the same phase. Beyond complete synchrony, however, networks of nonlinear oscillators can also display sophisticated spatiotemporal patterns, such as waves that travel across the network at different spatial frequencies (10–12). In neuroscience, traveling waves have recently been found in recordings from the visual cortex during active sensory processing (13). Specifically, in studying the visual cortex during active visual processing, we have found that a small visual stimulus evokes a wave of activation traveling outward from the point of input (14) and that natural image stimuli also evoke waves of activity traveling over an entire cortical region (15).

These waves traveling over individual cortical regions prompt an interesting computational question. Input from the eyes is organized into a “retinotopic map” (16): each cortical region in the visual system contains a complete map of visual space, because neurons at each point in an area receive feedforward input from only a small portion of visual space. From the perspective of the organization of single visual regions, one may then expect that visual processing is carried out locally. Specifically, one may expect a static image input to result in a static activity pattern, corresponding to how well local patches of the image drive the feature selectivity in each cortical region (17). The feedforward input, however, represents only approximately 5% of the inputs to a single cell in the visual cortex (18). While these feedforward synapses are strong, the dense, within-region recurrent connectivity makes up about 80% of connections a cell receives (18). We have recently found that this recurrent connectivity generates traveling waves in single regions of the visual cortex (14, 19), potentially overlaying the input to individual cortical regions with additional, internally generated dynamics. What could be the computational advantage of these internally generated traveling waves? Could they interfere with local cortical processing, or could they perhaps provide some computational benefit?

In this work, we demonstrate that networks of oscillators can perform object segmentation with traveling waves. We focus on oscillator networks with recurrent architecture, where nodes within the same layer can be connected. This recurrent architecture, which is consistent with the dense connectivity found in the visual cortex, is distinct from the standard feedforward networks (where nodes are arranged into layers with no intralayer connections) often employed for these tasks (4). Recurrent neural networks (RNNs) are considered relatively less often for object segmentation tasks, in part because they are more difficult to train than feedforward networks (20, 21). As in ref. 5, the network we consider is complex-valued, but with a recurrent structure as in ref. 9. This combination leads to a network that can dynamically generate spatiotemporal patterns of activity in response to image input, while also allowing for a precise analysis using a mathematical framework we have recently developed (22, 23).

These results open a different avenue in image segmentation by providing fundamental insight into how oscillator networks (6, 9) and complex-valued autoencoders (5) can be trained to perform object segmentation. These results also open possibilities for new algorithms and hardware-based implementations because the specific network we use is easy to implement directly in electronics. The mathematical analysis we introduce here may open new paths for understanding computations in RNNs, in particular for specific networks that have recently been found to have key advantages over Transformers in some long sequence prediction tasks (24, 25). Finally, these results also provide insight into visual processing in biological brains, by demonstrating a first computational example of why populations of neurons in a region of visual cortex might respond to a static stimulus with a dynamic activity pattern, as we have recently observed in experimental recordings.

## Results

We have recently developed a line of research that uses spectral graph theory to gain insight into the dynamics of recurrent oscillator networks (22). This mathematical approach allows predicting the wave patterns that result from the precise pattern of connections in a nonlinear oscillator network (23), which is a difficult problem in applied mathematics in general. Building on this analysis, we have found that a complex-valued neural network composed of these nonlinear oscillators can function as a dynamical reservoir to make simple video predictions (26).

In this work, we now introduce a simplified form of our previous network that can perform image segmentation while also being exactly solvable. The exactly solvable computation is made possible by combining insights derived from spectral graph theory, which allows us to design a network that will produce long transients sufficient for computation, and our mathematical analysis of nonlinear oscillator networks. With this combination, we design a complex-valued network with linear recurrent interactions that exhibits long transients in the amplitude of each node while also exhibiting meaningful evolution of the phases. This specific configuration leads to recurrent network dynamics that remain bounded during the transient, allowing for the image computation to occur while also being exactly solvable in a closed-form mathematical expression. We note that while these complex-valued dynamics could be represented by a network with real-valued states, the use of complex numbers greatly simplifies the equations used later in the mathematical analysis.

In the following sections, we first detail the architecture of the network we introduce in this work, which we term a complex-valued recurrent neural network (cv-RNN). We then demonstrate that this cv-RNN can segment objects in a variety of images, ranging from binary images with simple geometric objects to grayscale naturalistic images. Finally, we present a mathematical analysis of how the cv-RNN performs this segmentation, through studying the eigenspectrum of the matrix describing the complete system.

### Network Architecture.

**Fig. 1.**
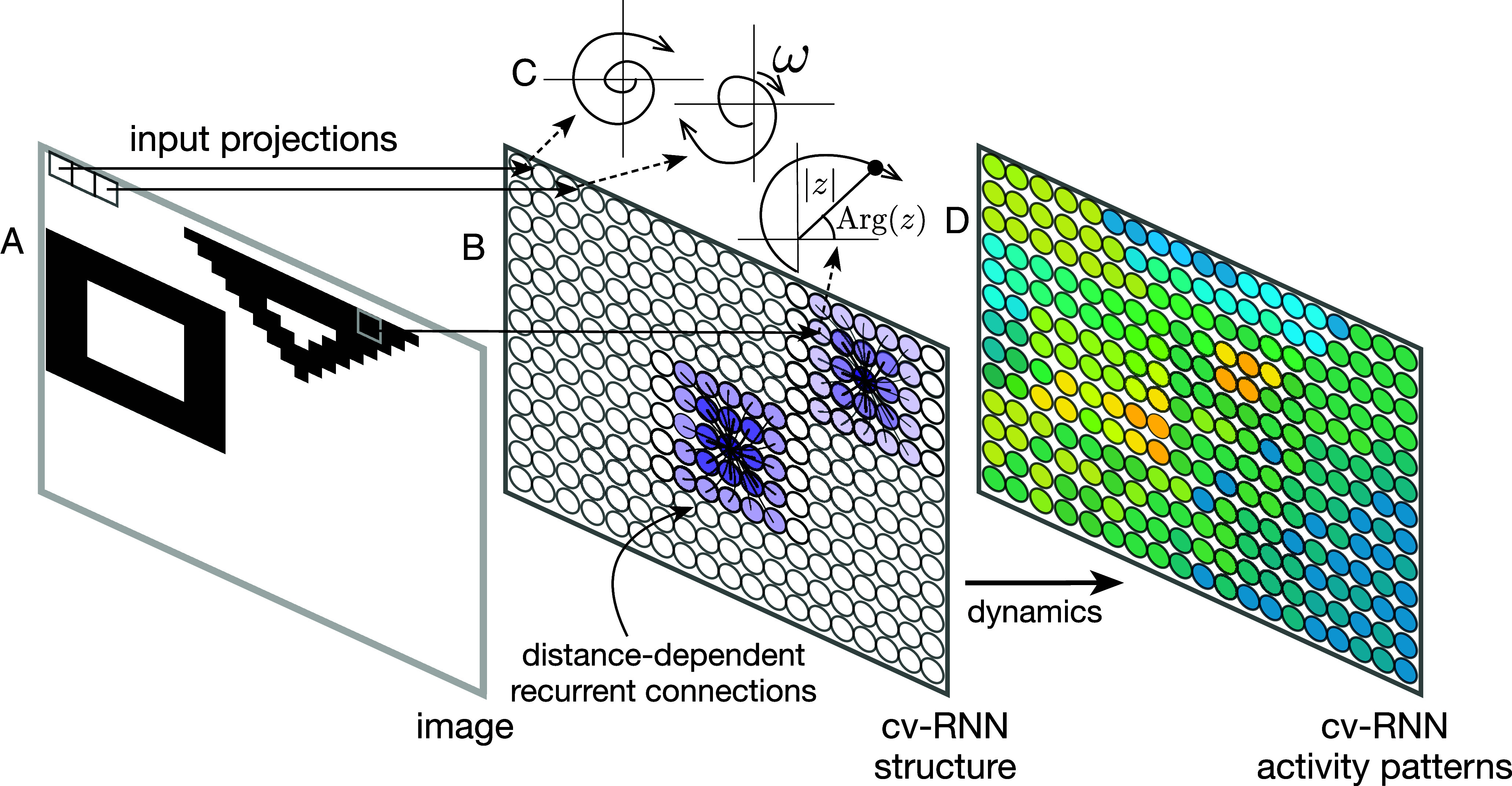
Schematic representation of the cv-RNN. (*A*) Input image. Each pixel projects to one node in the cv-RNN. (*B*) Nodes in the cv-RNN are arranged into a 2D sheet, where recurrent connection weights (purple) decrease as a Gaussian with distance between nodes Eq. **4**. (*C*) The activity of each node is described by a phase Arg(z) and an amplitude |z| in the complex plane. Inputs from image pixels modulate the natural frequency ω of the corresponding node. (*D*) Image inputs interact with the recurrent dynamics of the cv-RNN, to produce spatiotemporal patterns of activity in the network that can be used to segment images.

The cv-RNN is arranged on a two-dimensional square lattice with a side length of N nodes. Each node in the network receives input from one pixel of an image (Fig. 1). Nodes in the oscillator network are densely connected with their local neighbors (“distance-dependent recurrent connectivity,” Fig. 1), approximately following the connectivity that occurs in single regions of the visual cortex (18). We consider a specific dynamical equation for the evolution of this system of $N^{2}$ nodes:

[1] $$\begin{array}{cc} \dot{\psi_{i}}\left(t\right) & =\omega_{i}+\epsilon \sum_{j=1}^{N^{2}}a_{\mathrm{ij}}\left[\right.\sin\left(\psi_{j}\left(t\right)-\psi_{i}\left(t\right)\right)\\ & \;-\;\text{i}\;\cos\left(\psi_{j}\left(t\right)-\psi_{i}\left(t\right)\right)\left.\right], \end{array}$$

where $\psi_{i}\left(t\right)\in \;C\;$ is the state of node i at time t, $\omega_{i}\in \;R\;$ is the node’s intrinsic oscillation frequency, the matrix element $a_{\mathrm{ij}}\in \;R\;$ is the connection between nodes i and j, and ϵ∈R scales the strength of all connections. We note that throughout this paper, we consider i the imaginary unit, such that $\sqrt{-1}=\;\text{i}$, in contrast to i, which represents an index.

It is important to note that Eq. **1** bears similarity to the defining equation for networks of nonlinear oscillators (27), differing from the equations for a Kuramoto oscillator network by an additional imaginary component in the interaction term. We have recently demonstrated that this specific system displays the hallmark behaviors of synchronization that have been found in these oscillator networks (22, 23) and have developed a mathematical approach to explain the spatiotemporal dynamics that occur in these networks. By defining the change of variable,

[2] $$\begin{array}{c} \begin{array}{cc} x_{i}\left(t\right) & =e^{\;\text{i}\psi_{i}\left(t\right)}=e^{-\;\text{Im}(\psi_{i}\left(t\right))}e^{\;\text{i}\;\text{Re}(\psi_{i}\left(t\right))},\\ x_{i}\left(t\right) & =|x_{i}\left(t\right)|\;e^{\;\mathrm{Arg}\;\left[x_{i}\left(t\right)\right]}, \end{array} \end{array}$$

we find that Eq. **1** admits an exact solution (22, 28). Now, taking the system in discrete time, we can express the recurrence as

[3] $$\begin{array}{c} x\left(k+1\right)=\underset{B}{\underbrace{\left(\;\text{diag}(\;\text{i}\omega )+\epsilon A\right)}}x\left(k\right), \end{array}$$

in matrix form (*SI Appendix*, section I), where $A\in {\;R\;}^{N^{2}\times N^{2}}$ contains the connections in the network. Here, we input the image to the recurrent network by modulating the intrinsic frequency of each node. Specifically, each pixel of the image drives the intrinsic frequency $\omega_{i}$ of each node, with higher pixel intensities resulting in faster oscillation frequencies. The matrix $B\in {\;C\;}^{N^{2}\times N^{2}}$ describes the complete cv-RNN, where image inputs interact with recurrent connections to produce spatiotemporal patterns that allow segmentation. Because B contains information about both the recurrent connectivity and the input to the network, we refer to it as a “composite” matrix later in this work.

Connections in the recurrent layer have strength that decreases with their Euclidean distance $d_{\mathrm{ij}}$ between two nodes on the square lattice:

[4] $$\begin{array}{c} a_{\mathrm{ij}}=\alpha \;\exp\left(\frac{-d_{\mathrm{ij}}^{2}}{2\sigma^{2}}\right), \end{array}$$

where α∈R sets the peak strength of connections, and σ∈R controls how fast connection strength falls off with distance. The architecture of these connections sets the scale for local recurrent interactions in the cv-RNN, allowing nodes whose input pixels are nearby the opportunity to interact and create shared spatiotemporal patterns. Throughout this work, the cv-RNN starts with random initial conditions, with node amplitudes $|x_{i}\left(0\right)|$ distributed uniformly in the interval [0,1] and phases Arg[x(0)] uniform in [−π,π].

### The cv-RNN Creates Spatiotemporal Patterns Unique to Each Object in an Input Image.

**Fig. 2.**
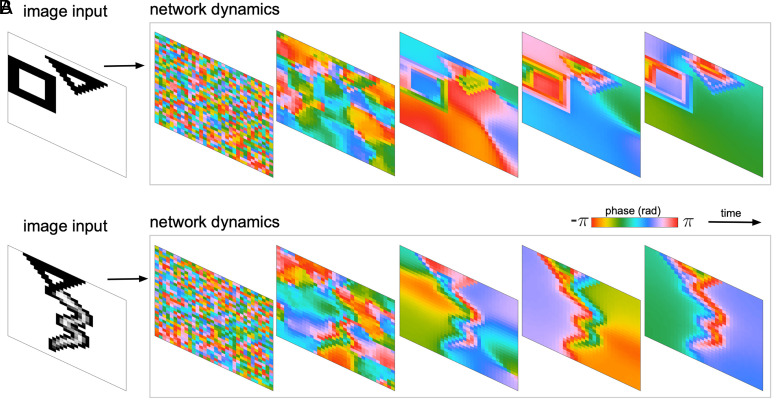
Spatiotemporal dynamics produced by the cv-RNN. (*A*) An image drawn from the 2Shapes dataset (see *Materials and Methods, Image Inputs and Datasets*) is input to the oscillator network by modulating the nodes’ intrinsic frequencies ω. The samples of the phase dynamics in the recurrent layer during transient time show that the nodes are imprinting the visual space by generating three different spatiotemporal patterns: one for the nodes corresponding to the background in the input space, one for the nodes corresponding to the square in the input image, and finally for the nodes corresponding to the triangle in the input space. (*B*) Image drawn from the MNIST&Shapes dataset is input into the dynamical system. Three different spatiotemporal patterns arise: one for the nodes corresponding to the background in visual input space, one for the nodes corresponding to the triangle in the input space, and finally for the nodes corresponding to the handwritten three-digit.

We first consider the cv-RNN with inputs drawn from a dataset used in recent work on complex-valued autoencoders (5), which have simple, binary-encoded geometric shapes. We study the cv-RNN dynamics on these simple geometric objects, and on combinations of geometric objects and MNIST digits, before moving to more complex inputs and naturalistic images. With an input containing a triangle and a square, the cv-RNN begins with random initial conditions, where the phases of the nodes are desynchronized (Fig. 2*A*, “network dynamics,” first panel). Interactions between nodes are captured by elements of the system matrix $b_{\mathrm{ij}}$, where the absolute value of the connection $|b_{\mathrm{ij}}|$ changes the strength of interaction between two nodes, and the phase of the connection $\;\mathrm{Arg}\;\left[b_{\mathrm{ij}}\right]$ changes their relative angle. These features of the connections are sufficient to drive traveling waves unique to the triangle, square, and image background (Fig. 2*A*, network dynamics; see also Movie S1). Importantly, the wave traveling over the background has a substantially lower spatial frequency than the waves traveling over each object in the image, separating the objects and background into two very different sets of spatiotemporal patterns. With the same set of recurrent weights A, and a new input image—this time containing a triangle and an MNIST numeral 3—the cv-RNN again produces unique waves traveling over the triangle, the numeral “3,” and the background (Fig. 2*B*). These results demonstrate that the cv-RNN can generate spatiotemporal patterns from its internally generated recurrent dynamics. We next tested whether these spatiotemporal dynamics, in combination with an unsupervised method for separating the individual phase patterns we have recently developed, could perform image segmentation.

### Object Segmentation Algorithm.

Having observed that recurrent interactions can produce traveling wave patterns unique to each object, we next developed an algorithm to segment objects using these dynamics. This algorithm uses a two-layer implementation of the cv-RNN, where the first layer separates image objects from the background. Briefly, after a fixed number of time steps, the dynamics in the first layer separate the objects in an image from the background. This separation then determines which nodes participate in the second layer, whose dynamics are run in order to segment the individual objects. In this way, the algorithm comprises a two-step approach to object segmentation, with each step solved through linear dynamics in a cv-RNN.

**Fig. 3.**
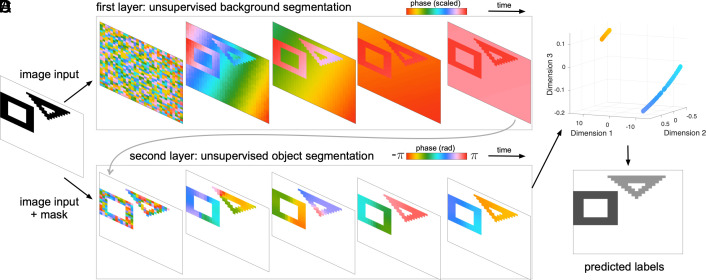
Object segmentation algorithm. (*A*) A first cv-RNN layer with broad spatial connectivity segments the image background. In this plot, samples of the phase dynamics (reshaped to a N×N grid) at each point in time show a unique phase for the nodes corresponding to the background in the visual input space. Pixels corresponding to foreground objects synchronize on a single phase distinct from the background. (*B*) After timestep k=60, nodes corresponding to background pixels are disconnected from the rest of the recurrent network in the second cv-RNN layer. Then, the second layer dynamics begins, where connections between nodes in the second layer create sophisticated spatiotemporal dynamics unique to each object. (*C*) The similarity projection in the low dimensional space for the phase dynamics generated in the second layer shows that the spatiotemporal patterns propagate through the nodes corresponding to the objects of the visual input. The phase patterns are separated into two different groups by the K-means algorithm. (*D*) Labels assigned to objects in the input by the K-means algorithm.

Connection patterns specific to each layer facilitate this process. In the first layer, the recurrent connections have a higher peak strength α and a broader spatial scale σ. The broad spatial scale of the recurrent connections, together with the different intrinsic frequencies driven by the input, creates a difference in the dynamics for nodes whose inputs have objects as input and for those nodes whose input is the background. With this architecture in the first layer, the phase dynamics converge to two different sets of synchronous nodes, with one set capturing the background and the other capturing the objects (Fig. 3*A*). The mean phase value across the network then separates nodes in objects from the background, which we then use to segment the background. In the second layer, nodes assigned to the background are disconnected from the rest of the recurrent layer, and the remaining recurrent connections have lower α and a smaller spatial scale σ. With this architecture in the second layer, the phase dynamics of the cv-RNN then display traveling waves that clearly separate the individual objects in the image (Fig. 3*B*). By conducting a comprehensive numerical study over network hyperparameters for two training images in a dataset of binary images with two objects (5), we were able to identify a single set of recurrent weights for layers 1 and 2 that generates clearly unique traveling wave patterns over image objects, across cases where the objects are in different positions in the image and at different relative distances. In terms of the connection between oscillator networks and the recurrent dynamics studied here, this set of weights corresponds to a network that generates a set of traveling waves useful for image segmentation, during a sufficiently extended transient period. We note that the range of σ generating successful segmentations will depend, to some extent, on the spatial scale of objects in the images; in future work, however, implementing several cv-RNNs in parallel, each with connectivity on different spatial scales, could facilitate segmenting objects on different spatial scales.

The only remaining step is to segment the phase patterns in the second layer into specific object labels. To do this, we use a method we have recently developed to find repeated spatio-temporal patterns in multisite neural recordings (29). Briefly, if $\phi_{i}$ represents the phase dynamics of node i for a set of time points T, then we can compute similarity $s_{\mathrm{jk}}$ between nodes j and k through the complex inner product:

[5] $$\begin{array}{c} s_{\mathrm{jk}}=\frac{1}{T}\left\langle \phi_{j},\phi_{k}\right\rangle , \end{array}$$

where T=|T|. By computing the similarity between the dynamics of each pair of nodes, we can then construct a similarity matrix $S\in {\;C\;}^{N^{2}\times N^{2}}$. Because S is complex-valued and Hermitian, its eigenvalues $\lambda_{1},\lambda_{2},...,\;\lambda_{N^{2}}$ are real-valued, and its eigenvectors $z_{1},z_{2},...,\;z_{N^{2}}$ have complex elements. Note that we will order the eigenvalues by decreasing absolute value, so that $|\lambda_{1}\left|\geq \right|\lambda_{2}\left|\geq ...\geq \right|\lambda_{N^{2}}|$. We then project the real part of S onto the real part of its leading eigenvectors:

[6] $$\begin{array}{c} P=\hat{S}\;\hat{Z}, \end{array}$$

where $\hat{S}$ is the element-wise real part of S, and $\hat{Z}$ is a matrix whose columns are the real part of eigenvectors $z_{1}$, $z_{2}$, and $z_{3}$. This projection defines a three-dimensional space that describes the similarity between phase dynamics. Each node in the cv-RNN becomes one point in this space, with node position determined by its relative similarity to the dynamics of the other nodes over time. Clustering the individual waves traveling over each object is straightforward in this three-dimensional similarity space (Fig. 3*C*). We then use a simple clustering algorithm to label the points in this space. The network dynamics in the cv-RNN create a set of points that are well separated in similarity space, as is visually apparent in Fig. 3*C*. We use K-means throughout this work, but note that the results do not depend on the specific choice of clustering algorithm. Further, the network dynamics in the cv-RNN are the key step in this process, as image segmentation is not successful when cv-RNN hyperparameters are unoptimal (*SI Appendix*, Fig. S2).

With this approach, the two-layer cv-RNN robustly segments objects in the set of inputs with two geometric objects in the test dataset (5). On average, 93% of pixels in 1,000 images of two nonoverlapping geometric shapes were correctly clustered and 86% in 1,000 images of three nonoverlapping geometric shapes (*SI Appendix*, section III), comparable to the range reported in previous work (5, 30, 31). These results demonstrate that the cv-RNN developed here enables generalization to inputs where objects are not in the same position of the image but can have rotation or translation.

### A Single Set of Recurrent Weights Segments Images across Datasets.

**Fig. 4.**
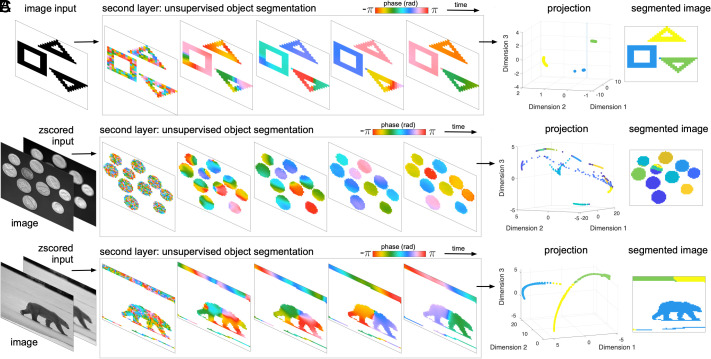
A single set of recurrent connection weights segments objects from simple images to naturalistic visual scenes. Image inputs are shown in the left column, and some samples of the phase dynamics in the cv-RNN are depicted in the middle column. Panel (*A*) contains an input with simple geometric shapes, panel (*B*) shows a naturalistic image input of coins on a dark background and panel (*C*) also shows a naturalistic image of a bear. Projection onto the eigenvectors of the similarity matrix separates the phase patterns in a three-dimensional space (second column from right, labeled “projection”). Labels assigned to objects in the input by the K-means algorithm are plotted in the right column.

Having developed a two-layer cv-RNN that can perform object segmentation in simple images, we next tested the cv-RNN for more complex inputs and natural images. Using the same set of recurrent weights identified above, we find that the cv-RNN can also perform object segmentation on these more sophisticated examples. The cv-RNN can successfully segment inputs with three or more distinct geometric objects in the image (Fig. 4*A*).

We next tested whether the cv-RNN, without modification, could also segment objects in natural images. We tested five natural images in total (Fig. 4 and *SI Appendix*, Fig. S4), including an image with ten coins (Fig. 4*B*) and an outdoor scene with a bear (Fig. 4*C*). We z-scored these images and input them directly into the cv-RNN, with the same set of recurrent weights derived from the simpler images. We find that, even with much more sophisticated inputs, unique traveling waves occur for each object in the second layer of the cv-RNN, allowing successful object segmentation in these natural images (Fig. 4, right column).

These results demonstrate the ability of the cv-RNN to generalize across inputs with varying numbers of objects, and even to novel visual inputs, without changing weights or hyperparameters derived from a few simple images. This adaptability demonstrates the breadth of inputs that can be handled by the recurrent dynamics in our approach. The ability of the cv-RNN to segment three objects in an image when previously optimized for only two objects demonstrates the utility of the orderly topographic layout of the network, where oscillators representing each pixel interact most strongly with nearby pixels, naturally generating wave patterns that propagate over individual objects. We note that the topography of the cv-RNN is linked to successful segmentation for objects in a specific range of spatial scales; however, by running several cv-RNNs in parallel, each with a different spatial scale of connectivity, this architecture could be generalized to work across object scales in future work.

**Fig. 5.**
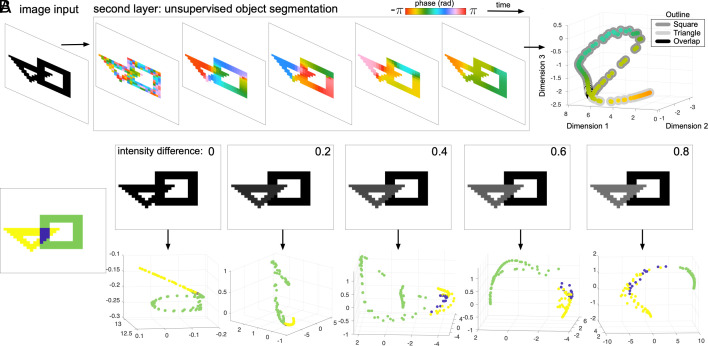
Segmentation of overlapping objects. (*A*) When objects in the input image overlap, the object segmentation algorithm separates the nonoverlapping sections into different objects. (3D plot at *Right*) Points in the projection are colored by the final value in the phase dynamics. Outlines for each point denote the object to which each point belongs in the ground-truth input. The points are arranged into two closed loops that meet where the pixels in the image overlap. (*B*) Small differences in the pixel intensities for each object separate the similarity projection for each object. (*Left*) Ground-truth labels for this case of partial overlap, with the triangle in yellow, the square in green, and pixels belonging to the overlap zone in purple. (*Top* row) Differences in pixel intensities for each foreground object range from 0 to 0.8 (*Top Right* corner of input images), and nodes in the overlap zone (purple nodes) receive the same input intensity as the triangle (yellow nodes). (*Bottom* row) Plotted is the similarity projection for each input case, with nodes in the projection colored according to which zone they belong in the input image. When the pixel intensities differ for the two objects, the pixels in the overlap zone are assigned the intensity of the triangle. As the difference in pixel intensity between the triangle and the square increases, the separation between clusters in the similarity projection grows. As in Fig. 4, all image segmentation is performed with the same set of recurrent weights and hyperparameters.

In these previous cases, the images considered contained sets of objects that were nonoverlapping. Phase dynamics within the second recurrent layer create unique traveling wave patterns that eventually converge to unique synchronized phases for each object. The interaction of the random initial conditions with the recurrent interactions in the second layer allows the network to generate different phase values for the nodes corresponding to different objects in the image input. However, an important question is whether this approach can also work when objects overlap to some extent. An example of this case is an image of a triangle and a square from the binary shapes dataset (5) where the two objects overlap. The pixels where the two objects do not overlap can then be segmented using the unsupervised phase similarity method (Fig. 5*A*, *Right*). The wave patterns traveling across each object lead to closed loops in the similarity space, with the oscillators for pixels in the overlap zone meeting at the intersection of the two loops (nodes with black outline, Fig. 5*A*). In this overlap zone, the pixels display an interesting behavior, as the two waves meet in the overlap zone, and these nodes then exhibit phases that are consistent with either spatiotemporal pattern. This ambiguity, however, only holds for the case where inputs are binary. In the case where pixel intensities differ slightly for each object, as expected for natural images in general, the phase similarity representation shows increasing separation for each object as the difference in intensity grows (Fig. 5*B*). These results hold over a range of inputs with partially overlapping objects (*SI Appendix*, section IV).

These results demonstrate that the cv-RNN produces unique spatiotemporal patterns that enable segmentation with our unsupervised phase similarity technique, even in the nontrivial case where objects in the input overlap. The cv-RNN tolerates substantial overlap before the phase patterns become indistinguishable (*SI Appendix*, section IV and Fig. S1). Finally, it is important to note that, while we do not consider segmentation for the case of complete object overlap here, and instead focus on the cases where objects can be separated based on information present in the individual input image, adding additional learning mechanisms to the cv-RNN in future work could allow the network to identify specific objects for segmentation, in addition to performing more sophisticated image processing tasks.

### The Exact Solution for the cv-RNN Allows Mathematical Analysis of the Segmentation Computation.

The results presented above demonstrate the cv-RNN can segment objects in input images ranging from simple arrangements of geometric objects to natural scenes. The optimal set of recurrent weights, then, is expressive enough to perform this image processing computation. In addition, the equation defining the cv-RNN can be exactly solved, in a closed-form mathematical expression. This solution provides an opportunity to understand precisely how the cv-RNN performs the computation.

To understand the connection between dynamics and computation in the cv-RNN, we can study the eigenspectrum of the composite matrix B. As noted above, this matrix contains information about both the structural properties of the cv-RNN and the input image. Specifically, the diagonal entries of B contain information about the input image, while its off-diagonal entries represent the network’s recurrent connectivity. As a result, the eigenvectors of B will change with each new input image to the cv-RNN. The dynamic nature of the eigenspectrum of B, in turn, allows us to understand how the network generates waves unique to each object and to perform image segmentation.

The spatiotemporal impact of the eigenvectors $v_{i}$ (for all i in the range $\left[1,N^{2}\right]$) of B can be understood in a precise manner, through a framework we have recently developed for networks of nonlinear oscillators (22, 23). In this framework, we considered networks of Kuramoto oscillators, which are studied to understand synchronization phenomena throughout nature (32). We then introduced a complex-valued system that could describe the Kuramoto network dynamics through application of specific matrix operations. This analysis, in turn, provides the underlying insight that the spatiotemporal patterns that will occur in the cv-RNN can be understood in terms of the phase of the eigenvectors (more specifically, $\;\mathrm{Arg}\;[{\left(v_{i}\right)}_{j}]$ for each element j of eigenvector i of the matrix B studied here) (23). This mathematical analysis, which was first developed for networks of Kuramoto oscillators, can also be applied to the simpler case of the linear cv-RNN dynamics in this work, and provides two specific insights. First, this analysis allows us to understand which configurations of the cv-RNN will generate long dynamical transients before diverging to large amplitudes (or zero), in terms of the eigenspectrum of B. As noted in introducing the architecture of the cv-RNN, this insight allows us to design connectivity structures that will admit transients sufficient for image segmentation to occur. Second, the analysis also allows us to precisely understand the traveling wave patterns that occur in the cv-RNN dynamics, which we will study in detail below.

**Fig. 6.**
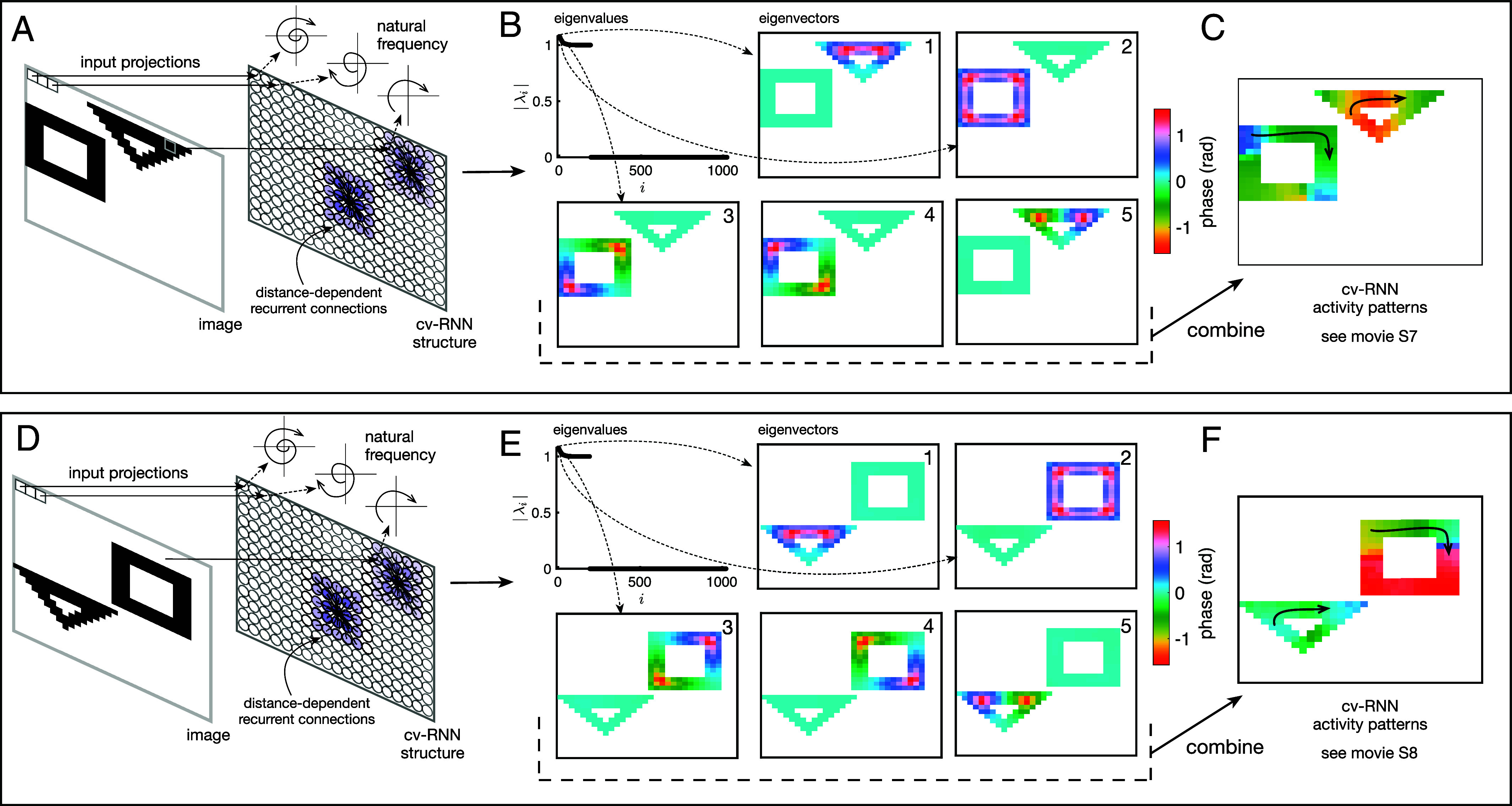
The eigenvectors of matrix B illustrate how the cv-RNN creates traveling wave patterns unique to each object. (*A*) An input image interacts with the recurrent dynamics specified by connections in the network by modulating the natural frequencies of each node. Due to the network’s topographic structure, nodes corresponding to nearby pixels in an image are more strongly connected. If these pixels are also part of the same object, the corresponding nodes will have similar natural frequencies, causing their dynamics to evolve in a similar way. (*B*) The interaction between image inputs and recurrent connections can be understood through eigendecomposition of this linear system. Here, eigenvalues are plotted in descending order of absolute value. The argument of the eigenvectors corresponding to the first five eigenvalues are also plotted. Each eigenvector specifies a wave pattern in the cv-RNN. Note that these patterns are object specific. (*C*) Dominant eigenvectors dictate the spatiotemporal patterns that appear in the cv-RNN, See Movie S7. (*D*–*F*) Same as *A*–*E* for a different input image. Note that the network connectivity is identical, but the differing input leads to different dominant eigenvectors for this input image, and thus different spatiotemporal patterns. See Movie S8. The precise interaction between specific inputs and the network structure can be studied through this framework, providing insight into how the same set of connections can produce very different activity patterns to segment a variety of images.

This understanding now allows us to study traveling wave patterns generated in the cv-RNN through the eigenvectors of B. The cv-RNN dynamics can be understood through the equations:

[7] $$\begin{array}{c} x(k)=B^{k}x(0)=\sum_{i=1}^{N^{2}}\underset{\mu_{i}(k)}{\underset{︸}{\lambda_{i}^{k}\left(r_{i}^{T}x(0)\right)}}v_{i}, \end{array}$$

where $\lambda_{i}$ are the eigenvalues associated with eigenvectors $v_{i}$ of B, $r_{i}^{T}$ are the rows of ${\left[v_{1}\cdots v_{N^{2}}\right]}^{-1}$, and coefficients $\mu_{i}\left(k\right)$, which depend on the initial conditions and eigenvalue, weight the contribution of each eigenvector. The spatiotemporal dynamics in the cv-RNN, which are generated by the linear combination of eigenvectors weighted by terms $\mu_{i}\left(k\right)$, can be understood in terms of traveling waves generated by each eigenvector $v_{i}$. A first image will serve as an example input to the cv-RNN (Fig. 6*A*). To illustrate the spatiotemporal impact of each eigenvector, we plot the argument of each element of the eigenvector at the oscillator corresponding to a pixel in the input image in color code (Fig. 6*B*). In this case, the top eigenvector of B exhibits phases that range from 0 to $\frac{\pi }{2}$ in the triangle and phases that are approximately 0 in the square (Fig. 6*B*, eigenvector 1). In terms of the cv-RNN dynamics, this generates a wave traveling in the triangle and promotes phase synchrony in the square (Fig. 6*B*, eigenvector 1). The next three eigenvectors generate a wave traveling clockwise on the square while promoting phase synchrony in the triangle (Fig. 6*B*, eigenvectors 2 to 4). Finally, the fifth eigenvector of B works to create a wave traveling clockwise on the triangle, by causing oscillators receiving input from pixels on the left side of the triangle to have earlier phases than oscillators on the right side of the triangle (Fig. 6*B*, eigenvector 5). Taken together, these eigenvectors work to create object-specific traveling waves (Movie S2) that, in turn, enable easy segmentation of the triangle and square in the phase similarity space.

When a new image input is applied to the cv-RNN, the new elements along the diagonal of B interact with the off-diagonal elements representing the internal connectivity in the cv-RNN to produce a new object-specific pattern of traveling waves. In terms of the eigenspectrum of B, a second image where the triangle is positioned below the square (Fig. 6*D*) results in eigenvectors of B generating similar phase offsets within the objects, but in the new positions for the triangle and square (compare Fig. 6*E* with Fig. 6*B*). This linear combination of eigenvectors, then, results in object-specific traveling waves for the second input image, again allowing easy labeling of each object.

The eigendecomposition analysis presentented above demonstrates how input images interact with recurrent connectivity to produce object specific waves in the cv-RNN. Segmenting the objects in an input image then becomes a matter of detecting and labeling specific waves in the network. Here, we implement this segmentation by clustering cv-RNN nodes that exhibit similar phase dynamics. We use phase similarity to measure the similarity between dynamical trajectories of nodes in the complex plane. Pairs of nodes with high similarity in phase evolution correspond to nodes that exhibit similar dynamics and thus to nodes that are part of the same wave or subpattern in the system dynamics. As a specific example, nodes that participate in a low spatial frequency wave traveling around a square object evolve differently compared to nodes participating in a high frequency wave traveling the opposite direction around a triangular object and are driven by distinct eigenmodes (Fig. 6 *B* and *E* and Movies S7 and S8). Eigendecomposition of the system thus provides a mechanistic understanding for how the interaction between input images and recurrent connections in the network drive the nodes to cluster in this way. Taken together, these results demonstrate a direct link between the eigenspectrum of B and the object-specific traveling waves underlying segmentation by the cv-RNN. Finally, we note that this decomposition indicates the cv-RNN dynamics can be approximated through a low-order truncation, using only a few eigenvectors and eigenvalues of the full matrix B (*SI Appendix*, section VI and Movie S6), which could substantially reduce the load for computing cv-RNN dynamics in practice.

Neural networks are widely considered “black boxes,” whose inner workings are difficult to understand. Current methods for explainable AI (33, 34) aim to provide heuristic rationales for the decisions made by a model but do not necessarily aim to reveal the model’s inner workings. By introducing a network that can perform a specific image processing computation (segmentation) while also being exactly solvable, we introduce a mathematical formulation that can go beyond heuristic insights into model behavior, providing a precise mathematical equation and closed-form solution enabling complete interpretability of the mechanisms used for the computations. In this way, the mathematical insights into the specific computation studied here may provide a more general path toward mathematical descriptions of neural network computation in future work.

## Discussion

In this work, we have introduced a new recurrent neural network, with complex-valued dynamics, that can perform object segmentation in a range of images with a single set of weights and that admits an exact solution. By focusing on transient dynamics and on connectivity regimes that lead to specific eigenvalue sets for the recurrent connectivity matrix A, we find that the cv-RNN can perform segmentation using the simplest linear dynamics possible. This system, in turn, admits an exact mathematical solution, which we can leverage not only to explain exactly how this network performs the computation of image segmentation, but also to design dynamics to achieve new computations in future work. In this way, this linear cv-RNN represents an opportunity to drastically simplify some neural network computations in computer vision and beyond. Further, these results represent an advance in explainable artificial intelligence (XAI). The ability to specify the dynamics leading to the image segmentation computation in a precise mathematical expression surpasses current techniques for explaining how neural networks make decisions. This mathematical approach may represent an important future direction for XAI research, specifically in introducing highly transparent and interpretable neural networks for computer vision and beyond.

Previous work has studied oscillator networks to segment images through groups of nonlinear oscillators that synchronize when they are part of the same object (9). Other studies have also employed dynamical systems for image segmentation, such as the research conducted in ref. 6, where the authors present an algorithm to find boundaries in natural images analogous to a spring-mass harmonic oscillator. In ref. 6, Belongie and Malik developed a link between standard image segmentation algorithms such as normalized cuts to the dynamics of harmonic oscillators, by mapping the normalized cuts directly onto the eigenvectors of a harmonic oscillator system. Their system corresponds to shaping the dynamics of a spring-mass oscillator network by changing connections using filtered versions of the input. Approaches such as this rely on shaping the connections in a network through a training procedure like gradient descent, which gradually adjusts the weights over the course of many image presentations. While these trained weights can successfully segment specific sets of images, they do not always generalize well to new types of inputs. Here, we take a different approach to shaping the network structure: with a grid search across a small subset of training images with two geometric objects, we find a specific, recurrent, and topographic connection scheme that can generalize surprisingly well to thousands of similar images, as well as images with additional objects and a handful of natural scenes. Finally, future implementations of the cv-RNN could employ standard algorithms such as backprop to train ϵ and σ. In this case, the parameters that would be learned would be substantially more constrained than in a standard RNN.

Further, while the current implementation of the cv-RNN falls in the middle of the range of computational complexity for image segmentation algorithms (*SI Appendix*, section VII), the ability to truncate dynamics using only a few eigenvectors further distinguishes the cv-RNN from deep networks and other intricate learning algorithms, where the network dynamics cannot be approximated in such a straightforward manner. Low-rank approximations for the cv-RNN dynamics could thus form the basis for princpled and efficient implementations of the cv-RNN dynamics. While we focus on the basic insights provided by the full recurrent dynamics of the cv-RNN and our mathematical analysis in the present work, the truncation approach could aid the deployment of the cv-RNN dynamics for image segmentation applications in future work. In addition, the current cv-RNN runs in pixel space, but the algorithm can be applied in more general contexts that could represent improvements over pixel space. For example, the cv-RNN could be run in a (likely smaller) latent space. The input to the cv-RNN, which modulates the natural frequencies of the nodes, need not be given by the exact values of the image pixels, as in our current implementation. Instead, the cv-RNN input could be an arbitrary function of the image input—like the output of a convolutional layer, for example. Running the cv-RNN in such a latent space represents a future path towards integrating the cv-RNN with deep networks and would allow the size of the cv-RNN to be significantly reduced compared to the input images. This approximation can drastically reduce the dimensionality of the system.

The results presented here provide unique insight into how the networks of oscillators proposed in refs. 6 and 9 can learn to segment objects in images ranging from simple geometric constructions to naturalistic inputs. Further, our results also provide insight into how complex-valued autoencoders recently introduced in ref. 5 learn to synchronize phases with each different object. In the end, our results provide a critical simplification: a complex-valued linear dynamical system can perform the segmentation computation in its transient dynamics, successfully segmenting a wide range of images with a single set of recurrent weights. This key simplification was made possible by combining insights derived from spectral graph theory with our mathematical analysis of nonlinear oscillator networks to design a linear cv-RNN that exhibits long transients in each node’s amplitude, while also exhibiting meaningful evolution of the phases. While amplitudes diverge in linear systems in general, the fact that the phase dynamics can meaningfully evolve while the amplitude dynamics remain bounded represents a potentially useful general property for computing with complex-valued linear systems. The technical approach we introduce here thus makes it possible to leverage the simplicity of a linear network while also keeping the dynamics bounded in a range that is useful for processing visual inputs. Further, the fact that one connectivity matrix allows the system to segment many images, without learning a new structure of network connections, reframes the problem of image segmentation into a single recurrent topographic architecture, which generates a set of traveling waves that are useful for this image segmentation task. We can then analyze how the network performs this computation through a direct mathematical analysis of the eigenspectrum of the resulting system matrix.

Recent interest in RNNs has centered on a deeper understanding of their underlying mechanisms and strategic design choices, particularly by incorporating complex-valued activations (24, 25). These efforts have shown that linear RNN layers can exhibit remarkable expressive power when coupled with multilayer-perceptron blocks. In fact, they have outperformed their nonlinear counterparts in tasks of long sequence prediction (24, 25). The results in this present work, however, demonstrate that linear cv-RNNs can perform sophisticated computations without additional processing layers, such as multilayer perceptrons. Our findings thus not only align with these recent works but also streamline the network architecture by showing that fully linear recurrent layers are sufficient for object segmentation, which is a central task in computer vision. The way the cv-RNN solves this problem is not susceptible to problems that often arise when training recurrent neural networks, such as the vanishing gradient problem (20, 35). Our results further allow mechanistic interpretability for these networks that greatly improves the understanding of their inner workings and could potentially contribute to the development of novel computational algorithms.

Taken together, these results demonstrate the utility of recent insights into oscillator network dynamics (22, 23) and their potential interdisciplinary application to computer vision tasks. The cv-RNN introduced here performs object segmentation through relatively simple network dynamics that can also be solved exactly. Because most segmentation algorithms require highly sophisticated training regimes, this approach has the potential to drastically reduce the computational burden of some image processing tasks. Further, in cases where reduced precision for segmentation can be tolerated, this cv-RNN admits a simple low-rank approximation that can be easily truncated at any order. The flexibility of this approach demonstrates, in an additional manner, the utility of having a comprehensive mathematical description for neural networks. We hope that this first example of a neural network that can both perform a nontrivial computer vision task and be solved exactly opens doors for application across domains while also leading to innovative new algorithms in the field of image segmentation.

## Materials and Methods

### Image Inputs and Dataset.

Image inputs are drawn from two binary datasets (2Shapes, 3Shapes) and one grayscale dataset (MNIST&Shapes). Both are obtained from ref. 5. We also consider a naturalistic grayscale image representing coins and several naturalistic grayscale images drawn from the (36) dataset. All images of geometric shapes are of size of 32×32 pixels, and the naturalistic image is 64×64. The 2Shapes dataset contains images with two randomly positioned objects (square and triangle), where some overlapping between objects can occur. Images from the 3Shapes dataset introduce a third randomly positioned object (another triangle in a different orientation). The MNIST&Shapes dataset combines an MNIST digit (37) with a randomly positioned shape in each image. The naturalistic images include animals, objects, and buildings.

### Network Connectivity and Synchronization Mechanism.

Starting from a random and asynchronous initial condition x(0), the following equation governs the dynamics of the amplitude and phase of state vector x(k) of a cv-RNN layer:

[8] $$\begin{array}{c} x\left(k+1\right)=\underset{B}{\underbrace{\left(\;\text{diag}(\;\text{i}\;\omega )+\epsilon A\right)}}x\left(k\right), \end{array}$$

where $A\in {\;R\;}^{N^{2}\times N^{2}}$ contains the connections in the network, and $\omega \in {\;R\;}^{N^{2}}$ is a vector that describes the intrinsic properties for the nodes in the network (in our work, ω contains the image inputs to the cv-RNN). Matrix $B\in {\;C\;}^{N^{2}\times N^{2}}$ describes the overall system matrix.

The synchrony mechanism between the node’s complex values results from the combination of the adjacency matrix and the perturbation introduced by the input, which gives rise to intricate spatiotemporal patterns during the transient dynamics. As noted in the main text, the adjacency matrix used in our approach is given by a distance-dependent connectivity rule, which is described by

[9] $$\begin{array}{c} a_{\mathrm{ij}}=\alpha \;\exp\left(\frac{-d_{\mathrm{ij}}^{2}}{2\sigma^{2}}\right), \end{array}$$

where α and σ∈R represent the lateral connections akin to the visual cortex.

The proposed segmentation algorithm is composed of two cv-RNN layers. The first layer represented by system matrix $B_{1}$ and connectivity matrix $A_{1}$ segments the spatiotemporal patterns generated by the nodes corresponding to the background in the image. Matrix $A_{1}$ is parameterized with a more significant gain α and SD σ compared to the second layer. Note that when the coupling parameter ϵ in Eq. **8** is set to a higher value, the length of the transient, where the cv-RNN displays sophisticated spatiotemporal dynamics, is shorter, as the network’s tendency to synchronize is accelerated. The parameter α in Eq. **9** influences the connectivity strength between nodes and increases the connectivity range in the final adjacency matrix. On the other hand, the parameter σ in Eq. **9** governs the overall inverse decay rate of the connectivity between the nodes. In this sense, the connectivity matrix of the first layer augments the network’s overall connectivity between nodes, meaning that further apart nodes have the same influence as close-apart nodes.

After the unsupervised background removal in the first layer, we continue to segment the rest of the foreground objects in the second layer, represented by the system matrix $B_{2}$. As discussed in the main text, we use the background removal to select the important connections in the cv-RNN to be used in the second layer. This leads to a new connectivity matrix $A_{2}$ where the connections with the background are disregarded, and the dynamics for the background nodes are not considered or evolved. The dynamics in the second layer start from a new random initial state $x_{2}\left(0\right)$. Matrix $A_{2}$ corresponds to a masked version of $A_{1}$ that does not propagate any dynamics for the background nodes. For the connectivity structure of matrix $A_{2}$ of the second layer, we decrease σ and α, which leads to more local connectivity.

**Fig. 7.**
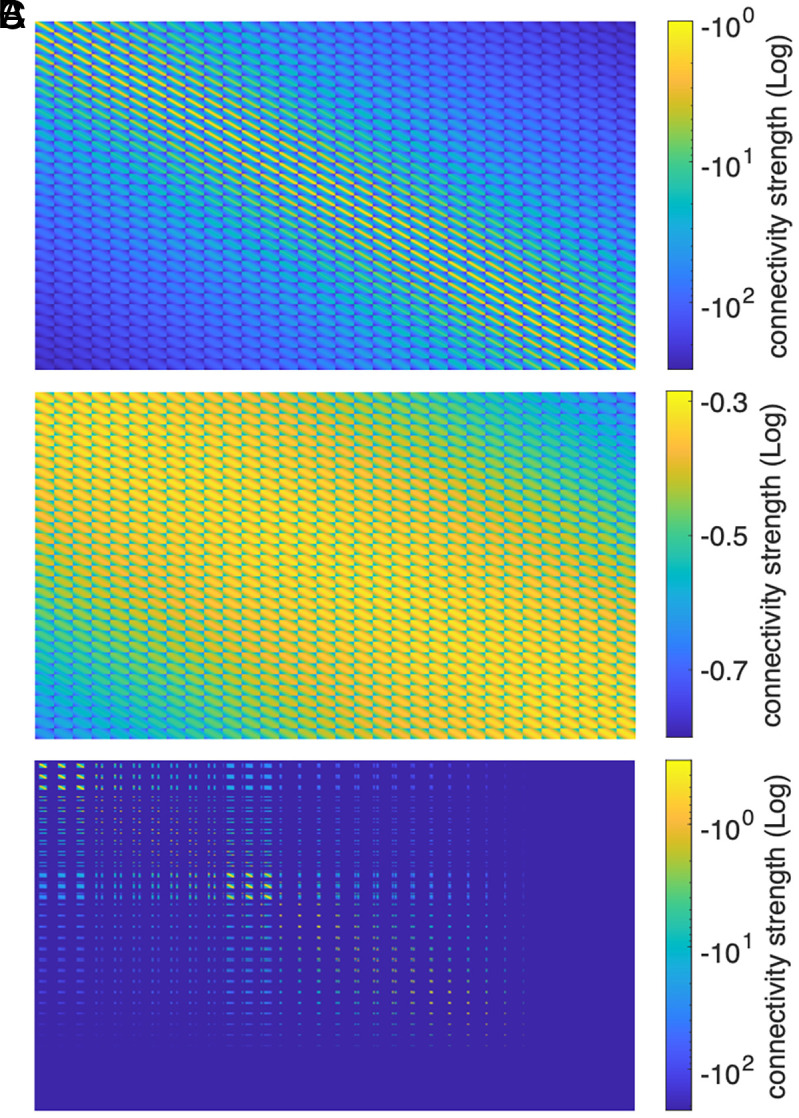
Adjacency matrix and cv-RNN connectivity. (*A*) Connectivity matrix A with parameters α = 0.51, σ = 0.0313, which was used in the case study of Fig. 2. (*B*) Connectivity matrix $A_{1}$ with parameters α = 0.5, σ = 0.9, responsible for driving the dynamics for background removal. (*C*) The masked connectivity matrix $A_{2}$ on logarithmic scale. The lines and columns of the connectivity matrix corresponding to the connections to the background nodes are assigned a value of zero and do not contribute to the dynamics of the object segmentation task.

Fig. 7*A* shows the structure of the connectivity matrix A for the single recurrent layer used to show that the spatiotemporal patterns are object-centric representations for Fig. 1 and Movie S1. In Fig. 7*B*, we can compare the structure of A with the structure of $A_{1}$, which was used in the first layer of the object segmentation approach to drive the dynamics for background removal. Fig. 7*C* shows the connectivity matrix $A_{2}$. Note that the lines and columns corresponding to the nodes of the background are masked, i.e., these nodes do not participate in the dynamics for the second layer.

### Network Exact Solution.

We can write the dynamics of the cv-RNN, described by Eq. **8**, in terms of the matrix B and its spectral properties:

[10] $$\begin{array}{c} x\left(k\right)=B^{k}x\left(0\right)=\sum_{i=1}^{N^{2}}\underset{\mu_{i}\left(k\right)}{\underbrace{\lambda_{i}^{k}\left(\;r_{i}^{T}x\left(0\right)\;\right)}}v_{i}, \end{array}$$

where $\lambda_{i}$ is the eigenvalues associated with the eigenvector $v_{i}$ of B and $r_{i}^{T}$ are the rows of ${\left[v_{1}\cdots v_{N^{2}}\right]}^{-1}$.

Because the cv-RNN dynamics can be written in terms of B and its eigenvalues and eigenvectors, we can express the spatiotemporal patterns that emerge in the network’s dynamics as a linear combination of the eigenmodes. Importantly, this is valid at all times, including during the transient period. The contribution of each mode i at time k is expressed by $\mu_{i}\left(k\right)$. Furthermore, it is important to note that the eigenvalues of B are complex. With this, we note that $\mu_{i}\left(k\right)$ rotates and changes its amplitude in the complex plane, and the combinations of specific modes lead to the emergence of sophisticated spatiotemporal patterns in the cv-RNN. Finally, this mathematical description is valid for both the first and second layers in our object segmentation process, where the properties of $B_{1}$ and $B_{2}$ shape the respective eigenvalues and eigenvectors for each case.

It is important to emphasize, however, that because a linear complex-valued equation governs the cv-RNN dynamics, the asymptotic behavior of the system is trivial: either the amplitude of the nodes’ dynamics diverges to infinity or decreases to zero. Before the asymptotic behavior is reached, however, the cv-RNN displays sophisticated spatiotemporal dynamics over long transients, as shown throughout this paper. Further, we can control the lifetime of the transient dynamics by modulating the eigenvalues of B using the connectivity parameters ϵ, and σ. With this, we obtain a neural network that offers an exact mathematical description of its dynamics in terms of the spectral properties of the system, while still being able to display sophisticated dynamics during transient, which we then use to perform image segmentation.

## Supplementary Material

Appendix 01 (PDF)

Movie S1. For *A* − *α* = 0.08, *σ* = 0.0313.

Movie S2. For *A* − *α* = 0.08, *σ* = 0.0313.

Movie S3. For *A* − *α* = 0.08, *σ* = 0.0313.

Movie S4. For *A*_1_ − *α* = 0.5, *σ* = 0.9, For *A*_2_ − *α* = 0.5, *σ* = 0.0313, *T* = 141 − 181. *σ* = 0.0313, *T* = 121 − 141.

Movie S5. For *A*_1_ − *α* = 0.5, *σ* = 0.9, For *A*_2_ − *α* = 0.5, *σ* = 0.0313, *T* = 141 − 181. *σ* = 0.0313, *T* = 121 − 141.

Movie S6. *A* − *α* = 0.08, *σ* = 0.0313.

Movie S7. For *A*_1_ − *α* = 0.5, *σ* = 0.9, For *A*_2_ − *α* = 0.5, *σ* = 0.0313.

Movie S8. For *A*_1_ − *α* = 0.5, *σ* = 0.9, For *A*_2_ − *α* = 0.5, *σ* = 0.0313.

## Data Availability

Open-source code have been deposited in GitHub (https://mullerlab.github.io/). Previously published data were used for this work (5, 36).
