# Supplementary material for: Image segmentation with traveling waves in an exactly solvable recurrent neural network
Source: Proc Natl Acad Sci U S A. 2025 Jan 3;122(1):e2321319121. doi: 10.1073/pnas.2321319121 (PMC11725882; doi:10.1073/pnas.2321319121)
Supplement: Supplementary file 1 — Appendix 01 (PDF) [file pnas.2321319121.sapp.pdf]

Supplementary Material - Image segmentation with traveling waves in an exactly solvable recurrent neural network

Luisa H. B. Liboni,[1,2,3,4,*] Roberto C. Budzinski,[1,2,3,4,*] Alexandra N. Busch,[1,2,3,4,*]
Sindy Löwe,[5] Thomas A. Keller,[6] Max Welling,[6] and Lyle E. Muller[1,2,3,4]
[1]*Department of Mathematics, Western University, London, ON, Canada*
[2]*Western Institute for Neuroscience, Western University, London, ON, Canada*
[3]*Western Academy for Advanced Research, Western University, London, ON, Canada*
[4]*Fields Lab for Network Science, Fields Institute, Toronto, ON, Canada*
[5]*AMLab, University of Amsterdam, Amsterdam, Netherlands*
[6]*UvA-Bosch Delta Lab, University of Amsterdam, Amsterdam, Netherlands*

I. DISCRETE-TIME RECURRENT COMPLEX-VALUED DYNAMICAL SYSTEM

We start from the continuous-time nonlinear complex-valued dynamical system described by

$$\dot{\psi}_i(t) = \omega + \epsilon \sum_{j=1}^{N^2} a_{ij} \left[\sin\left(\psi_j(t) - \psi_i(t)\right) - \mathrm{i}\, \cos\left(\psi_j(t) - \psi_i(t)\right) \right], \tag{S1}$$

where $\psi_i(t) \in \mathbb{C}$ represents the state of a node, $\omega \in \mathbb{R}$ specifies the frequency at which the nodes' dynamics evolve. The elements $a_{ij} \in \mathbb{R}$ determine the connections between nodes i and j and are scaled by the coupling parameter $\epsilon \in \mathbb{R}$.

By Euler's identity, Eq. (S1) can be rewritten as

$$\dot{\psi}_i(t) = \omega - \mathrm{i}\, \epsilon\, e^{-\mathrm{i}\psi_i(t)} \sum_{j=1}^{N^2} a_{ij} e^{\mathrm{i}\psi_j(t)}. \tag{S2}$$

We now multiply Eq. (S2) by $\mathrm{i}\, e^{\psi_i}$ to obtain

$$\mathrm{i} e^{\psi_i(t)} \dot{\psi}_i(t) = \mathrm{i}\, \omega\, e^{\psi_i(t)} + \epsilon \sum_{j=1}^{N^2} a_{ij} e^{\mathrm{i}\psi_j(t)}. \tag{S3}$$

and define the change of variables

$$x_i(t) = e^{\mathrm{i}\psi_i(t)}, \qquad \dot{x}_i = \mathrm{i} e^{\mathrm{i}\psi_i(t)} \dot{\psi}_i(t).$$

with which Eq. (S3) becomes,

$$\dot{x}_i(t) = \mathrm{i}\, \omega\, x_i(t) + \sum_{j=1}^{N^2} a_{ij}\, x_j(t). \tag{S4}$$

Finally, Eq. (S4) can be written in matrix form as

$$\dot{\boldsymbol{x}}(t) = \left(\mathrm{diag}(\mathrm{i}\, \boldsymbol{\omega}) + \epsilon \boldsymbol{A}\right) \boldsymbol{x}(t). \tag{S5}$$

We then consider the discrete-time version for Eq. (S5), which leads to

$$\boldsymbol{x}(k+1) = \underbrace{\left(\mathrm{diag}(\mathrm{i}\, \boldsymbol{\omega}) + \epsilon \boldsymbol{A}\right)}_{\boldsymbol{B}} \boldsymbol{x}(k), \tag{S6}$$

where $k \in \mathbb{Z}^+$ is a positive integer.

---

[*] These authors contributed equally

II. CONSIDERATIONS ON AMPLITUDE DYNAMICS

Because we are considering a linear complex-valued network, the nodes' amplitude $|x_i(k)|$ in Eq. (S6) naturally diverges to infinity or decreases to zero in asymptotic time. However, it is essential to notice that, for transient time, the amplitude and phase dynamics of the cv-NN display sophisticated behavior, which we leverage to perform calculations, more specifically, object segmentation.

Modulating the eigenvalues of $\boldsymbol{B}$ in Eq. (S6) can extend the transient time, resulting in the phase dynamics evolving nontrivially while the amplitudes remain finite, which we call feasible period. Therefore, in the feasible period, the amplitudes are still numerically bounded, and therefore, calculations are numerically feasible. The phase dynamics simulations depicted in Figure 2 of the main text are shown in Movie 1 and Movie 2, where it is clear that object-centric spatiotemporal patterns emerge and imprint each object, enabling further segmentation. Movie 3 further shows the amplitude dynamics for the same test case used in Movie 1.

III. SEGMENTATION PERFORMANCE

Although performance for object segmentation is not the primary goal of this paper, the general segmentation algorithm we propose aims to help us better understand the dynamics of the complex-valued dynamical system and draw a baseline for the robustness of this object-centric representation. As mentioned, object-centric representations are obtained after segmenting the background in the first layer and without any training mechanism. The K-means algorithm is applied to the similarity low-dimensional representation to obtain clusters of nodes with similar dynamics.

By applying the segmentation approach for forty timesteps $T = 40$ of the dynamics, we obtain an accuracy of 93% of pixels on 1000 images drawn from the 2Shapes dataset. Furthermore, 86% of pixels were correctly clusterized in 1000 images drawn from the 3Shapes dataset. Both analyses considered nonoverlapping configurations. Neighboring object configurations were also excluded from the testing dataset. These performances underscore the object-centric representation's remarkable efficiency, obtained without any training mechanism and with a very low computational burden.

IV. CONSIDERATIONS REGARDING OVERLAPPING CASES

As discussed in the main text (Figure 5), the nodes in the overlapping area co-participate in both spatiotemporal propagation that arises in the phase dynamics. This result shows that, in that region, the objects overlap, and one can retrieve the exact overlapping location from the pixel space. For naturalistic images, where different objects have slightly different pixel intensity values, the spatiotemporal patterns have been shown to be unique to each object.

We now extend our analysis to those images where different objects have the same pixel intensity. In Figure S1, we depict the similarity projection of the overlapping cases from the 2Shapes dataset, in which the foreground square and triangle intensity are given the value of 1, with different overlapping percentages. Therefore, the objects are nonseparable in the input space.

As one would expect, the more significant the percentage of overlap, the more difficult it is to cluster the spatiotemporal patterns into different object-specific representations. Given that no training mechanism is used, the spatiotemporal patterns obtained in these overlapping cases were remarkable.

V. MECHANISTIC INTERPRETATION OF THE CV-RNN AND THE ROLE OF THE HYPERPARAMETERS

The cv-RNN dynamics can be understood through the equations:

$$\boldsymbol{x}(k) = \boldsymbol{B}^k\, \boldsymbol{x}(0) = \sum_{i=1}^{N^2} \underbrace{\lambda_i^k \left(\boldsymbol{r}_i^T \boldsymbol{x}(0) \right)}_{\mu_i(k)} \boldsymbol{v}_i, \tag{S7}$$

where λ_i are the eigenvalues associated with eigenvectors $\boldsymbol{v}_i$ of $\boldsymbol{B}$, $\boldsymbol{r}_i^T$ are the rows of $[\boldsymbol{v}_1 \cdots \boldsymbol{v}_{N^2}]^{-1}$, and coefficients $\mu_i(k)$, which depend on the initial conditions and eigenvalue, weight the contribution of each eigenvector.

This complex-valued network is, in fact, a system of oscillators, a connection we have recently developed in depth [1]. In this previous work, we developed an operator-based approach where the dynamics of real-valued Kuramoto

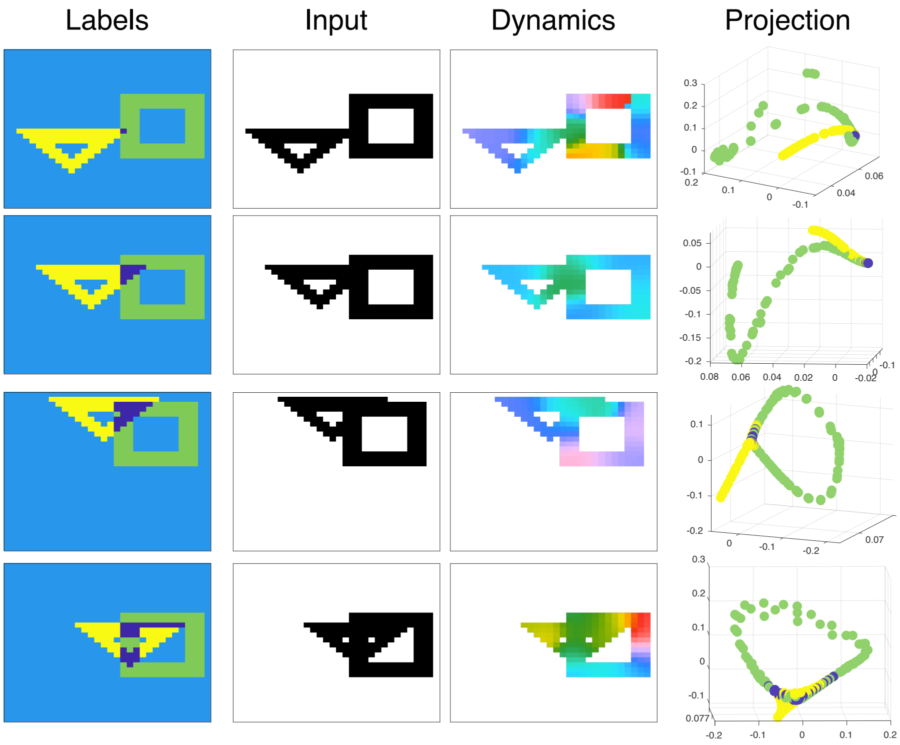

Figure S1. An input with non-disjoint foreground figures with a higher overlap percentage. The overlapping nodes can be identified as sharing two clusters.

oscillators could be matched exactly by using two operators to evolve a complex-valued system: the first is a linear operator, which rotates the oscillators in the complex plane; the second is a nonlinear operator, which normalizes the amplitudes of the nodes, so that the dynamics in the argument of the complex-valued system precisely match individual trajectories of the original, real-valued Kuramoto system [2]. Here, we leverage the mathematical insights developed along this line of research to introduce a new network that can perform computations while also being exactly solvable. To do this, we now drop the second, nonlinear operator, to study a system with sophisticated dynamics governed fully by a linear operator. It is these linear dynamics, specifically when looking at the phase values of the oscillators, that underlie computation in the cv-RNN.

A. Mechanistic Interpretation

We analyze the recurrent dynamics in the cv-RNN through the connection to our analysis of oscillator networks. Nodes in the cv-RNN are complex-valued oscillators whose natural frequencies are modulated by input images. These inputs interact with the recurrent dynamics specified by the topographic connectivity of the network to produce the spatiotemporal patterns used for image segmentation. In practice, nodes receiving input from pixels within delimited regions (similar pixel intensities and near to eachother) tend to synchronize. Moreover, propagations will occur because of the phase differences between different delimited areas.

These dynamics can be understood through our eigendecomposition analysis of the composite matrix $\mathbf{B}$, which contains the input image in diagonal entries, and captures the recurrent connectivity scheme in off-diagonal entries. Eigendecomposition can thus reveal the mechanism underlying segmentation in the cv-RNN, by explaining the interaction between image inputs and the underlying recurrent dynamics of the system. Image inputs to the cv-RNN shift the spectrum of the composite matrix $\mathbf{B}$, changing the contribution of eigenmodes in the system, and thus shaping the phase patterns that emerge.

Consider for example a network with local connectivity (Fig. S2b, left column). In this case, nodes are weakly affected by their neighbors, and the system does not present a prominent leading eigenvector. In practice, spatiotemporal dynamics occur with too fine a granularity to properly segment the objects (Fig. S2c, left). On the other hand, a network with stronger, global connectivity will quickly synchronize due to a leading eigenvalue with very large modulus. In this case, objects cannot be segmented because the network is fully synchronized (Fig. S2c, right).

However, when image inputs are combined with optimal recurrent connectivity, the eigenspectrum of the system shapes object-specific waves in the cv-RNN (Fig. S2b,c, middle). Eigendecomposition of the system thus provides a mechanistic understanding for how the interaction between input images and recurrent connections in the network drive the nodes to cluster into groups with separable, object-specific spatiotemporal dynamics.

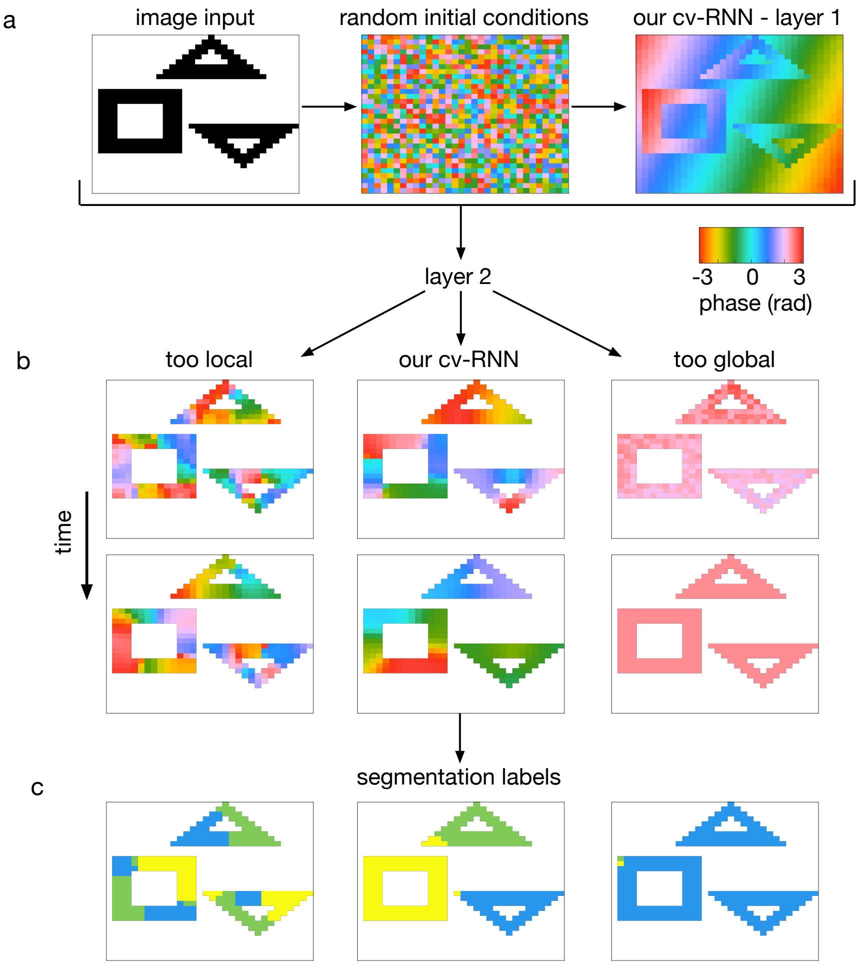

Figure S2. **Example of the influence of network connectivity on segmentation.** **(a)** We begin by inputting an image into layer 1 of our cv-RNN to obtain the mask for the three objects. We then manipulate the connectivity scheme in layer 2 to investigate the resulting segmentation. **(b)** Our cv-RNN (middle column) results in object specific waves. When connectivity is too local (left column), the spatiotemporal patterns that appear are choppy and local in space. When connectivity is too global (right column), the system quickly synchronizes (corresponding to a very large modulus first eigenvalue). **(c)** The segmentation labels resulting from the spatiotemporal patterns in each network are plotted. The local network assigns pixels within each object to many different clusters, while the more global network assigns all objects to the same cluster. Both of these results are as expected from the type of dynamics displayed.

B. The role of the hyperparameters

The hyperparameters in the cv-RNN are ϵ and σ, which control the peak strength and spatial extent of recurrent connections, respectively. These two hyperparameters have a clear physical interpretation, which we can understand through the connection with our analysis of coupled oscillators. From this perspective, cv-RNN nodes with stronger coupling will tend to quickly synchronize in phase, within regions determined by the spatial extent of recurrent connections, and nodes with weaker coupling will tend to synchronize much more slowly. Consistent with our understanding of coupled oscillator dynamics, then, ϵ controls, in general, the time to synchronize in the network. σ, as well, has

an equally straightforward physical interpretation, as it will control the spatial extent of recurrent interactions in the network. The specific point we find in (ϵ, σ) space, then, corresponds to a network that will generate traveling waves on the general spatial scale of objects in the image, during a sufficiently extended transient period. (See Fig. S2 for an example of how varying these parameters affects segmentation.)

C. Tuning Hyperparameters

To tune ϵ and σ, we conducted a grid search using only two images from the dataset of images with two geometric shapes. Considering only two example images at this point renders even a systematic search over the two-dimensional parameter space fast and effective. Further, even considering only two images for the tuning, we find an impressive amount of generalization: the cv-RNN that segments these two images then generalizes directly to the rest of the datasets considered in this work, including images with two or three shapes, combinations of shapes and MNIST digits, and the handful of natural images we studied. The ease of fine-tuning the hyperparameters in our approach is supported by the simple interpretation of the cv-RNN as a network of coupled oscillators able to generate object specific wave patterns.

VI. LOW-RANK SPATIOTEMPORAL RECONSTRUCTION

It is well-known that the eigenvectors of linear dynamical systems shape the resulting dynamics. Therefore, the linear combination of eigenvectors generates the spatiotemporal patterns that arise during the transient dynamics of the cv-NN. To show this, we use the eigenanalysis as exhibited in Figure 5 of the main text. As discussed, a finite number of eigenvalues of $\boldsymbol{B}_2$ have noticeably significant amplitudes with phases ranging from 1.20 to 1.57 rad, and the arguments of the eigenvectors are shaping bits and pieces of objects with a phase of π rad. Therefore, a low-rank reconstruction of the dynamics in Eq. (S6) can be obtained.

$$\boldsymbol{x}(k) = \boldsymbol{B}^k\,\boldsymbol{x}(0) = \sum_{i=1}^{n} \underbrace{\lambda_i^k(\,\boldsymbol{r}_i^T\boldsymbol{x}(0)\,)}_{\mu_i(k)}\boldsymbol{v}_i, \tag{S8}$$

where the linear combination of the eigenvectors $\boldsymbol{v}_i$ is truncated for a low-rank order n, $\boldsymbol{r}_i^T$ are the rows of $[\boldsymbol{v}_1 \cdots \boldsymbol{v}_{N^2}]^{-1}$, and $\mu_i(k)$ denotes the contribution of each eigenvector.

The contributions are dependent on the initial condition and also on the eigenvalues. Each contribution will rotate the phases of the nodes at each timestep k, giving rise to the spatiotemporal dynamics.

VII. COMPUTATIONAL COMPLEXITY

The computational complexity of the cv-RNN in inference time is $\mathcal{O}(\mathcal{T}N^4)$ where $\mathcal{T}$ is the number of timesteps of recurrent dynamics, and N^2 is the dimension of the state vector. The evolution of recurrent dynamics is followed by a clustering algorithm of complexity $\mathcal{O}(\mathcal{L}N^2)$, with $\mathcal{L}$ accounting for linear factors related to the number of clusters, dimension of projection, and time to convergence. Image segmentation algorithms, such as normalized cuts shi2000normalized, watershed approaches [3], graph cuts [4], and convolutional networks [5] operate directly on pixel values within a local neighborhood and have computational complexity $\mathcal{O}(N^2)$ to $\mathcal{O}(N^6)$. This range of computational complexity demonstrates that, in the current implementation, the cv-RNN algorithm falls within the middle of the range of computational complexities for other image segmentation approaches. It is important to note, in addition, that the cv-RNN requires tuning only two hyperparameters specifying recurrent connectivity in the network, reducing the overall computational burden compared to training deep learning architectures, which require tuning many hyperparameters for successful performance. Finally, since the cv-RNN (equation 5 of the main text) is a linear-time-invariant recurrence (as with SSMs), by using a parallel associative scan algorithm [6], it is possible to parallelize this over sequence length, to get $\mathcal{O}(\log(\mathcal{T}))$ complexity for sequence length, which would result in $\mathcal{O}(\log(\mathcal{T})N^4)$ complexity. The linearity of the system is the feature enabling a scan like this, making the system easily scalable.

VIII. EXTRA RESULTS

Figure S4 shows segmentation results for other naturalistic images selected from the BSDS500 dataset [7]. The images were resized to 64×64 pixels size and z-scored. It is clear that the object segmentation algorithm can remove

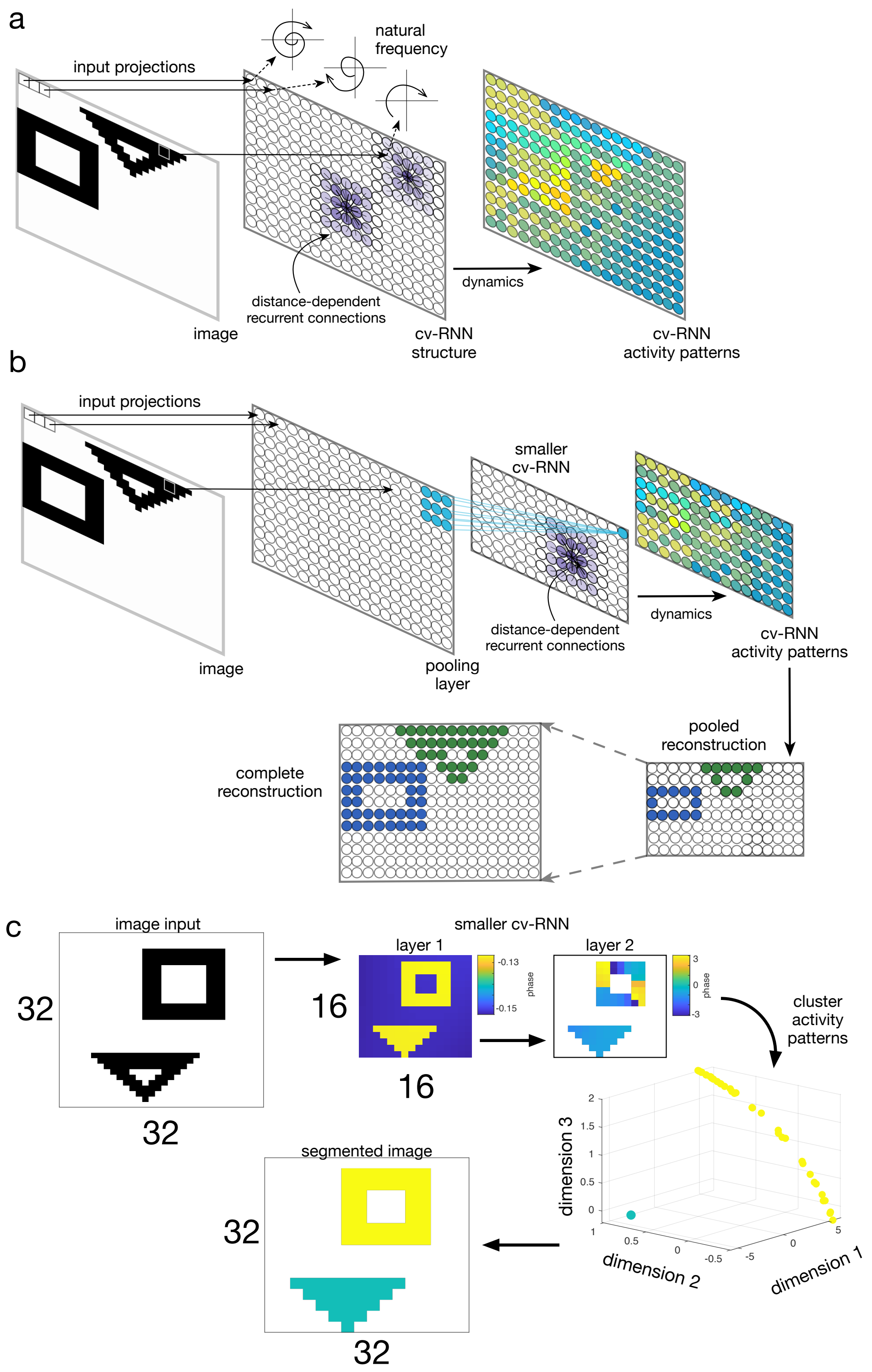

Figure S3. **(a)** Each pixel in an input image projects to one node in the cv-RNN. The cv-RNN network is a 2D sheet with distance-dependent connectivity, which is uniform across nodes. Each node is an oscillator in the complex plane. Input images influence the natural frequency of the corresponding node. Image inputs interact with the recurrent dynamics of the cv-RNN, to produce spatiotemporal patterns of activity in the network that can be used to segment images. **(b)** A pooling layer can be added so that an input image may project to a smaller cv-RNN. The activity patterns in the cv-RNN can still be used to segment the image, by first clustering the cv-RNN nodes, then reversing the operation performed by the pooling layer. **(c)** An example of one image segmented by a 2-layer cv-RNN with half the number of nodes as the pixels in the image.

significant portions of the background and make segmentation possible through its dynamics. For nonoverlapping objects in the naturalistic scenes, the network achieved the same very peculiar propriety: nodes corresponding to pixels of a particular object are locked up to unique phase values during some timesteps of the feasible transient regime.

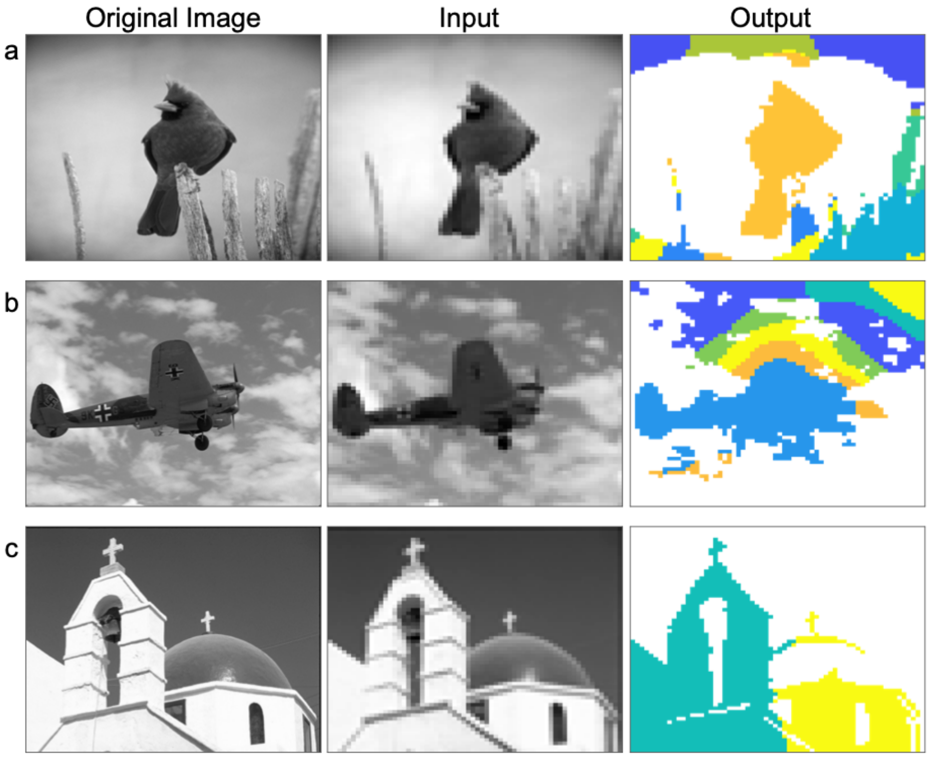

Figure S4. The original image (first column), input image (second column), and output (third column) of K-means clustering are plotted for 3 example naturalistic images.

IX. MOVIES

A. Movie 1

In this movie, we use Eq. (S6) to simulate the complex-valued linear system with a visual input image from the 2Shapes dataset containing a triangle and a square (same input used in Figure 2a of the main text). The black pixels are assigned to -1 and the white pixels to 1. The movie shows the phases $\text{Arg}[\boldsymbol{x}(k)]$. At the beginning of the movie, we capture the phase's random initial condition $\text{Arg}[\boldsymbol{x}(0)]$. Closer nodes, which correspond to closer pixels, are strongly connected since the connectivity matrix is given by the decaying Gaussian rule in Eq. (4) from the main text. One can observe that the nodes corresponding to the background and the distinct foreground objects are being pushed by the $\text{diag}(\text{i}\boldsymbol{\omega})$ to oscillate in different intrinsic frequencies towards the end of the movie. This oscillation phenomenon agrees with what happens in continuous-time oscillators modeled Eq. (S5). The rationale behind this correspondence is linked to the modification caused by the diagonal encoding of the visual input, which leads to changes in the intrinsic frequencies of individual nodes.

B. Movie 2

Movie 2 shows the segmentation accomplished by the network's arguments for image input from the MNIST&shapes dataset, where a triangle and a handwritten number three are combined, as shown in Figure 2b of the main text. We use the same connectivity parameters as the ones used in Movie 1. The digit is grayscale values to capture boundary

effects. Note that the first pattern in the grid is being propagated through the nodes corresponding, in the input space, to the triangle. Another unique spatiotemporal pattern generated by the dynamics is propagating within the nodes related, in the input space, to the background, and, lastly, the third unique pattern that propagates is related to the nodes corresponding, in the input space, to the handwritten digit. Note that the different spatiotemporal patterns imprint the objects through their different progression in time.

C. Movie 3

Movie 3 shows the amplitude dynamics of the state vector $\boldsymbol{x}[k]$ reshaped into a $N \times N$ grid for the cv-NN in the same conditions as considered in Movie 1. The initial value for the amplitude vector is $|\boldsymbol{x}(0)| = \mathbf{1}$.

Given that this system is not stable, the amplitudes are shown to grow with k. We can perform image segmentation with the phase dynamics. However, as shown in Movie 3, the amplitude dynamics of the cv-NN are non-trivial during the transient time. Although we have not explored the amplitude dynamics in this paper, we expect to see more specialized algorithms arising in the future, which could, by applying a nonlinear operator, constrain the amplitudes and then leverage its information to improve object segmentation.

D. Movie 4

In this movie, we show the first step of the background removal for the coin's naturalistic visual input. In this input, the coins are considered to have z-scored grayscale intensity. In $k = 60$, the background is removed by disregarding the nodes with a higher frequency on the phase distribution and masking them so that they do not account for the second layer's system dynamics.

The second layer dynamics begins from a random phase condition for the unmasked nodes. Now, the phases of the nodes corresponding only to the foreground objects will progress. In the case of nonoverlapping objects, the nodes synchronize their phases to a unique value for each object while maintaining the background phase static. For completely binary inputs with a masked background, the rationale of why the nodes synchronize to a unique value for each object resides in the fact that the network is given a random starting point, which gives a stochastic difference for the phase synchronization for each object. In the case of grayscale ranges for the foreground objects and with the masked background, given that a continuum exists across the pixels within the same object, synchronization will happen in a small range of phases for each object.

E. Movie 5

In the left panel of movie 5, we show the spatiotemporal patterns generated for the case of overlapping objects from Figure 5 of the main text. In the right panel, we show the spatiotemporal patterns of the case of a 2Shapes image with a higher percentage of pixel overlap. We can see that the dynamical system cannot synchronize the nodes uniquely corresponding to each object because of the overlapping parts. However, the spatiotemporal patterns can again imprint each object, corroborating that the spatiotemporal patterns are unique propagation throughout the nodes mapped to each particular object and further separable. Because the inputs are not considered different objects in the raw image, the segmentation performance decreases with the amount of overlapping pixels.

F. Movie 6

In this movie, the same system used in Figure 6 of the main text is simulated through the linear combination of the six eigenvectors corresponding to the sixth most significant eigenvalues ($i \in [1, 6]$ in Eq. (S8)). We show that the segmentation can be achieved by using only a few numbers of eigenvectors to approximately reproduce the spatiotemporal pattern. These results demonstrate that the dynamics within a period of interest can be calculated very efficiently using low-rank approximations in their calculations. Therefore, note that by having matrix $\boldsymbol{B}_2$ obtained after masking $\boldsymbol{B}_1$, which is known apriori, one can easily calculate a few eigenvalues to generate approximately the spatiotemporal patterns by using just a few combinations of eigenvectors.

G. Movie 7

This movie corresponds to panel a of Figure 6 in the main text. The input image is depicted, followed by the eigenvectors corresponding to the ten most significant eigenvalues. The dynamics of the cv-RNN are then played.

H. Movie 8

This movie corresponds to panel b of Figure 6 in the main text. The input image is depicted, followed by the eigenvectors corresponding to the ten most significant eigenvalues. The dynamics of the cv-RNN are then played.

X. COMPUTATIONAL DETAILS AND PARAMETERS

In all cases, the connection architecture of the cv-NN is given by a distance-dependent lattice (Eq. (4) in the main paper) with $\epsilon = 1.0$, $\phi = 0$. The other relevant parameters can be found below:

- Main text

  Figure 2a,b: For $\boldsymbol{A} - \alpha = 0.2$, $\sigma = 0.0312$.

  Figure 3b: For $\boldsymbol{A}_1 - \alpha = 0.5$, $\sigma = 0.9$, $T = 141 - 181$.

  Figure 3c: For $\boldsymbol{A}_2 - \alpha = 0.5$, $\sigma = 0.0313$, $T = 141 - 181$.

  Figure 4a,b,c: For $\boldsymbol{A}_1 - \alpha = 0.5$, $\sigma = 0.9$, For $\boldsymbol{A}_2 - \alpha = 0.5$, $\sigma = 0.0313$, $T = 141 - 181$.

  Figure 5a,b,c: For $\boldsymbol{A}_1 - \alpha = 0.5$, $\sigma = 0.9$, For $\boldsymbol{A}_2 - \alpha = 0.5$, $\sigma = 0.0313$, $T = 121 - 141$.

  Figure 6: For $\boldsymbol{A}_1 - \alpha = 0.5$, $\sigma = 0.9$, For $\boldsymbol{A}_2 - \alpha = 0.5$, $\sigma = 0.0313$, $T = 121 - 141$.

- Supplementary material

  Figure S1: For $\boldsymbol{A}_1 - \alpha = 0.5$, $\sigma = 0.9$, For $\boldsymbol{A}_2 - \alpha = 0.5$, $\sigma = 0.0313$, $T = 141 - 181$.

  Figure S2: For $\boldsymbol{A}_1 - \alpha = 0.5$, $\sigma = 0.9$, For $\boldsymbol{A}_2 - \alpha = 0.5$, $\sigma = 0.0313$, $T = 141 - 181$.

  Movie 1, 2, 3: For $\boldsymbol{A} - \alpha = 0.08$, $\sigma = 0.0313$.

  Movie 4, 5: For $\boldsymbol{A}_1 - \alpha = 0.5$, $\sigma = 0.9$, For $\boldsymbol{A}_2 - \alpha = 0.5$, $\sigma = 0.0313$, $T = 141 - 181$. $\sigma = 0.0313$, $T = 121 - 141$.

  Movie 6: $\boldsymbol{A} - \alpha = 0.08$, $\sigma = 0.0313$.

  Movie 7, 8: For $\boldsymbol{A}_1 - \alpha = 0.5$, $\sigma = 0.9$, For $\boldsymbol{A}_2 - \alpha = 0.5$, $\sigma = 0.0313$

XI. CODE AVAILABILITY

An open-source code repository for this work is available on GitHub: http://mullerlab.github.io.

[1] L. Muller, J. Mináč, and T. T. Nguyen, Algebraic approach to the kuramoto model, Physical Review E **104**, L022201 (2021).
[2] R. C. Budzinski, T. T. Nguyen, J. Đoàn, J. Mináč, T. J. Sejnowski, and L. E. Muller, Geometry unites synchrony, chimeras, and waves in nonlinear oscillator networks, Chaos: An Interdisciplinary Journal of Nonlinear Science **32**, 031104 (2022).
[3] J. B. Roerdink and A. Meijster, The watershed transform: Definitions, algorithms and parallelization strategies, Fundamenta informaticae **41**, 187 (2000).
[4] Y. Boykov and V. Kolmogorov, An experimental comparison of min-cut/max-flow algorithms for energy minimization in vision, IEEE transactions on pattern analysis and machine intelligence **26**, 1124 (2004).
[5] K. He and J. Sun, Convolutional neural networks at constrained time cost, in *Proceedings of the IEEE conference on computer vision and pattern recognition* (2015) pp. 5353–5360.
[6] E. Martin and C. Cundy, Parallelizing linear recurrent neural nets over sequence length, arXiv preprint arXiv:1709.04057 (2017).
[7] D. Martin, C. Fowlkes, D. Tal, and J. Malik, A database of human segmented natural images and its application to evaluating segmentation algorithms and measuring ecological statistics, in *Proc. 8th Int'l Conf. Computer Vision*, Vol. 2 (2001) pp. 416–423.
